# De novo main-chain modeling with MAINMAST in 2015/2016 EM Model Challenge

**DOI:** 10.1016/j.jsb.2018.07.013

**Published:** 2018-07-31

**Authors:** Genki Terashi, Daisuke Kihara

**Affiliations:** a Department of Biological Sciences, Purdue University, West Lafayette, IN 47907, USA; b Department of Computer Science, Purdue University, West Lafayette, IN 47907, USA

**Keywords:** Cryo-EM, Electron microscopy, Protein structure modeling, CryoEM Model Challenge, Main-chain trace, Map interpretation, MAINMAST, Mean shifting algorithm, Minimum spanning tree, Rosetta, confidence score

## Abstract

Protein tertiary structure modeling is a critical step for the interpretation of three dimensional (3D) election microscopy density. Our group participated the 2015/2016 EM Model Challenge using the MAINMAST software for a de novo main chain modeling. The software generates local dense points using the mean shifting algorithm, and connects them into Cα models by calculating the minimum spanning tree and the longest path. Subsequently, full atom structure models are generated, which are subject to structural refinement. Here, we summarize the qualities of our submitted models and examine successful and unsuccessful models, including 3D models we did not submit to the Challenge. Our protocol using the MAINMAST software was sometimes able to build correct conformations with 3.4–5.1 Å RMSD. Unsuccessful models had failure of chain traces, however, their Cα positions and some local structures were quite correctly built. For evaluate the quality of the models, the MAINMAST software provides a confidence score for each Cα position from the consensus of top 100 scoring models.

## 1. Introduction

Recent technical improvements in cryo-electron microscopy (cryo-EM) have led to a rapid increase in macromolecular structures determined by cryo-EM (Frank, 2017), particularly those determined at a near atomic resolution (e.g. 4 Å or better). The statistics at EMDB (Patwardhan, 2017; Velankar et al., 2016) show that EM maps at 4 Å or better represent the fastest growing category among five resolution levels shown in the statistics (4, 6, 8, 10, 15 Å or worse) (https://www.ebi.ac.uk/pdbe/emdb/statistics_num_res.html/). From 2014 to 2017 this high resolution portion of the deposited maps in the EMDB increased its share of the total database by 92%, rising from 5.3% to 10.2%, nearly doubling in that time.

When an EM map is obtained, structure modeling of biomolecules, including proteins and nucleotides, in the map is a critical step for interpreting the map density. Various structure modeling techniques have been developed which are designed for maps of certain resolution ranges (Esquivel-Rodriguez and Kihara, 2013). Types of structure modeling tools include those used for atomic structure building originally developed for X-ray crystallography (Terwilliger et al., 2008), identifying main-chain conformations in a map (Baker et al., 2012a; Chen et al., 2016; Frenz et al., 2017; Wang et al., 2015), refining structure models (Afonine et al., 2018; DiMaio et al., 2009; DiMaio et al., 2015; Kirmizialtin et al., 2015; Trabuco et al., 2008), fitting known structures to density maps (Esquivel-Rodriguez and Kihara, 2012; Lopez-Blanco and Chacon, 2013; Miyashita et al., 2017; Woetzel et al., 2011; Wriggers and Birmanns, 2001), and identifying local structures in medium resolution (e.g. 6–10 A) maps (Baker et al., 2007; Jiang et al., 2001). Although structure modeling tools have been improving to keep pace with the rapid progress in microscopy instrumentation on 3D map reconstruction techniques (Hohn et al., 2007; Punjani et al., 2017; Scheres, 2012; Tang et al., 2007), modeling tools still have substantial room for improvement.

To critically evaluate 3D map construction and protein structure modeling techniques, EMDataBank is hosting community-wide challenges for the EM community. Following the first challenge meeting in 2010 (Ludtke et al., 2012), EMDataBank hosted two challenges in 2015/2016, the Map Challenge and the Model Challenge, for evaluating and discussing protocols and results for single particle reconstructions and for methods and results of building protein structure models, respectively. In the Model Challenge, submitted models were evaluated in one of the four modeling categories: 1) optimization of the current models; 2) fitting of known structures to maps; 3) ab initio model building; and 4) other types. Our group participated in the third category, ab initio model building. The Model Challenge consisted of eight target macromolecules with maps of a reported resolution ranging from 2.2 to 4.5 Å. The targets were released on October 14, 2015 and the deadline for the model submission was on June 17, 2016. The subsequent evaluation meeting was held on October 6–8 2017 at Stanford University, California, USA.

Our group has submitted ten models each for four targets using a de novo main-chain tracing software, MAINMAST (MAIN chain Model tracing from Spanning Tree), developed by us (Terashi and Kihara, 2018). Compared to other existing de novo modeling software (Chen et al., 2016; DiMaio et al., 2015; Frenz et al., 2017), MAINMAST is unique in that it does not refer to known structures, generates and ranks multiple structure models, and provides confidence levels of each residue positions by examining consensus among generated models. The modeling procedure using MAINMAST is fully automated and requires no manual parameter tuning or human intervention.

Here, we summarize and analyze the quality of the structure models of four target maps we submitted to the 2015/2016 Model Challenge. In addition to the submitted models, we also discuss models that were built for the other four target maps but not submitted to the Model Challenge. In addition to the protocol we used in 2016, we compare different components of structure refinements. A t the end of this report, we also show the confidence score of predicted models, which correlated well with the accuracy of their Cα positions.

## 2. Materials and methods

### 2.1. Model Challenge targets

Eight EM maps from EMDB were specified as targets in the 2015/2016 Modelling Challenge (http://challenges.emdatabank.org/?q=model-challenge-targets) (Table 1). As indicated, the target EM maps were published in the literature and were released at EMDB with fitted structures by the authors. Although fitted structures by authors were available, we modeled protein structures only from the density maps and did not refer to the author-fitted structures during the modeling since we participated in the ab initio modeling category to test our software, MAINMAST. However, as a preprocessing of maps before applying MAINMAST, we segmented EM maps according to the fitted structures in each map so that a map region only include a single chain. This process was needed since the current version of MAINMAST assumes that there is only a single protein chain in a map. For each density map, a single subunit (the A chain) was manually segmented from a whole density map using UCSF Chimera’s “zone tool” using the PDB structure as the reference.

### 2.2. The modeling protocol using MAINMAST

**Fig. 1.**
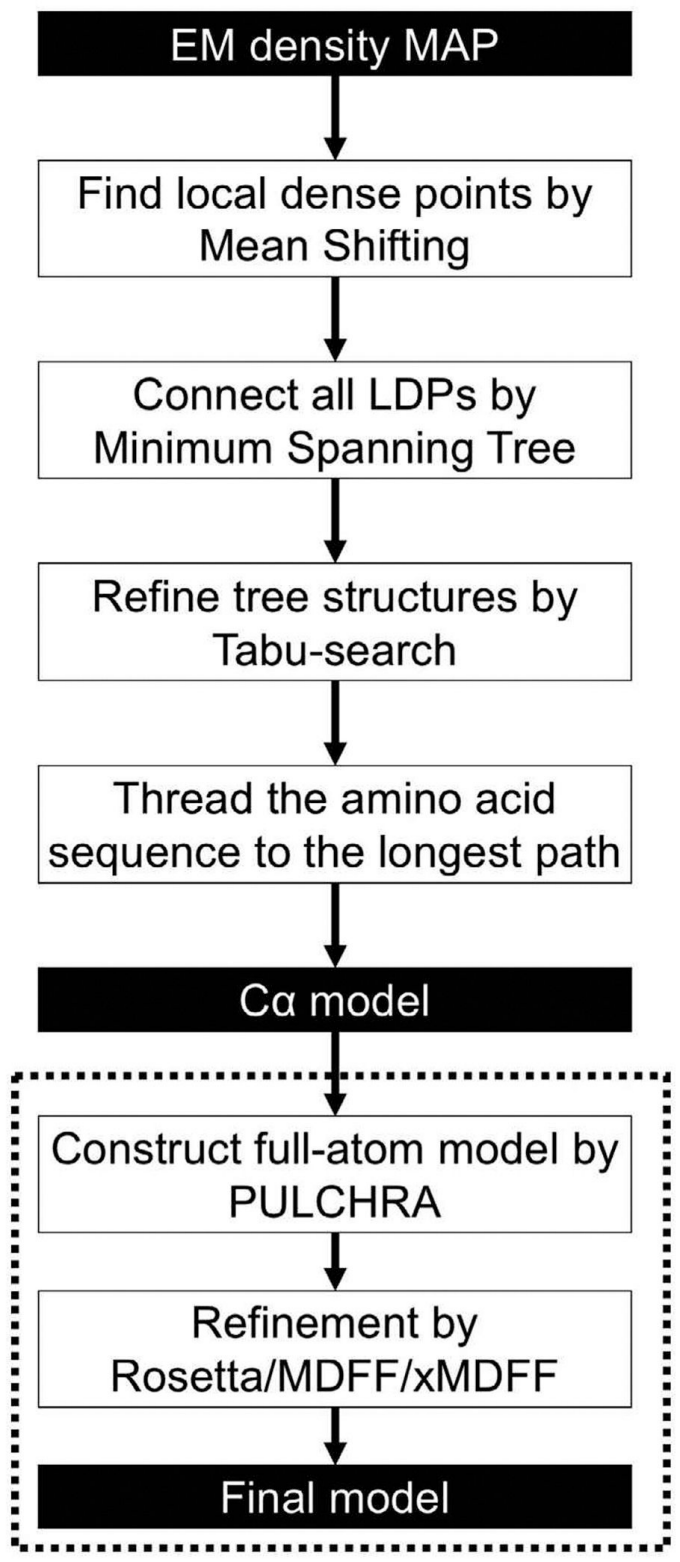
The flowchart of our modeling procedure using MAINMAST. First, points with a high local density are identified in the query EM map using the mean shift algorithm. Identified local dense points are connected by minimum spanning tree (MST). Using tabu search, the initial MST is refined. Next, the amino acid sequence of the protein is mapped on the longest path in the tree by matching the volume of amino acids to the density of the local dense points (threading). Trees were generated with different combinations of parameters that control the local dense point identification step, the tree refinement step, and the sequence mapping result, which results in over a few thousand Cα models. The Cα models are then ranked with the density-volume matching (threading) score. The 500 top-scoring Cα models are selected, which are subject to full-atomic structure building with PULCHRA and structure refinement with Rosetta, MDFF, or xMDFF. Finally, the 500 full-atom models are ranked by the scoring function of the refinement method used and the top 10 scoring models were submitted to the Model Challenge.

MAINMAST is a de novo main-chain structure modeling program for EM maps with resolutions of approximately 4–5 Å or better (Terashi and Kihara, 2018). Refer to the original paper for details of the algorithm. M AINM AST directly traces local dense regions of a map and does not refer to any known structures or structural fragments. M AINM AST consists of five steps (Fig. 1). In the first step, M AINM AST identifies local dense points (LDPs) in a density map using the mean shifting algorithm (Fukunaga and Hostetler, 1975). The implicit assumption is that a density observed in a map is the sum of Gaussian density functions that originate from atoms in the map. The density *k* of a position that originates from a grid point locating at a distance of *d* is defined as $k\left(d\right)=\exp\left(-1.5{\left\|\frac{d}{\sigma }\right\|}^{2}\right)$, where σ is set to 1.0. The total density of a position is the sum of the Gaussian-weighted densities from neighboring grid points. The mean shift algorithm starts from a set of grid points in the map that have a density value above a threshold value and iteratively move them toward local maxima until convergence is reached. The purpose of using mean shift is to perform local clustering to identify representative dense points. The number of LDPs is usually much more than the number of residues in the target protein. Typically, the number of clusters is about 40% of the number of heavy atoms of the underlined protein in the map.

In the second step, a minimum spanning tree (MST) is constructed that connects all LDPs. MST is a graph structure that connects all vertices with the minimal total weight of edges without forming cycles. It was found that the main-chain of the protein is well covered by the MST because the number of points is large enough so that neighboring points are found in a short proximity to one another.

In the third step, the obtained tree structure is refined iteratively. The obtained MST needs further refinement because in many cases the MST does not contain a perfectly correct path that corresponds to the main-chain of a target protein. Usually the longest path in the MST captures a large fraction of the correct main-chain trace, but there are several erroneous connections. To refine the tree structure, M AINM AST uses tabu search (Glover, 1986). Å tabu search attempts to explore a large search space by keeping a list of moves that are visited recently and thus are forbidden (a tabu list). During the tabu search, a tree structure was evaluated by the sum of the lengths of the top 100 longest paths in the tree. In each iteration of a tabu search, an existing edge is deleted from the tree and a new edge is added to maintain the tree structure.

In the fourth step, the longest path is identified in a tree. Then, local densities along the path is matched with the expected density of amino acids in the target protein sequence (i.e. amino acids with a large/small side-chain would be mapped to a position with a relatively high/low density on the path, respectively) using dynamic programming. This process assigns Cα positions of the target protein on the path. Since a protein sequence has a direction, the target sequence is mapped in two directions along the path. This sequence mapping process is similar to threading-type protein structure prediction methods, where a protein sequence is mapped to a protein main-chain model by considering compatibility of each amino acid in the sequence to local structural environment of positions in the model (Chen and Kihara, 2011; Skolnick and Kihara, 2001). The above steps are repeated with various parameter combinations, and generates over ten thousand models.

Finally, top 500 models ranked by the threading (sequence-path mapping) score are subject to the full-atom building using the PULCHRA program (Rotkiewicz and Skolnick, 2008). PULCHRA is a program that builds a full atom protein structure model from a reduced protein representation, which was originally developed for protein structure prediction. Subsequently, models were refined either by the ROSETTA refinement protocol (DiMaio et al., 2009), MDFF (McGreevy et al., 2016; Singharoy et al., 2016), or xMDFF (McGreevy et al., 2014) and ranked by the refinement protocol, i.e. the Rosetta Free Energy (RFE) in the case of Rosetta and the potential energy with a map fitting term in the case of MDFF or xMDFF. For MDFF and xMDFF, a parameter called g-scale, which balances the density map fitting term and a molecular mechanics force field, was set to 0.5. The detailed setting used for MDFF and xMDFF is provided in Supplemental Data. The top 10 scoring models by the refinement protocol were submitted to the Model Challenge. The models with high M AINM AST threading scores are sometimes rejected by the refinement protocol. Our modeling protocol is fully automated and free from manual parameter fitting or intervention. The protocol automatically tries several combination of the parameters to generate various models, and ranks them with the scores mentioned above. Refer to the original paper for further details (Terashi and Kihara, 2018).

MAINMAST is similar to the Pathwalking program (Baker et al., 2012b; Chen et al., 2016) in the basic concept that a protein structure model is built from an EM map by connecting identified local dense points in the map. The main differences between the two methods are the w ay points are identified and how they are connected. Pathwalking start with identifying points (which are called pseudo-atoms) that correspond to Cα positions of the target protein. Initially, more points may be identified, but they are reduced to the number of amino acid residues in the target protein by k-mean clustering. Thus, the traveling salesman problem solver is an appropriate choice for connecting the pseudo-atoms into a single chain. On the other hand, MAINMAST starts from many local dense points, which are usually much more than the number of amino acids in the target protein. The points include side-chain positions that have a large density value. Therefore, the points are connected into a minimum spanning tree, which allows branches. Other differences include that Pathwalking explicitly uses detected secondary structure information to adjust pseudo-atom positions. MAINMAST uses a threading approach to align the protein sequence onto a path in the minimum spanning tree.

In the results section, we discuss the accuracies of the models that were originally submitted to the Map Challenge as well as models generated for this work using the current version (ver. 2017) of MAINMAST. The differences between the 2016 version and the current version of MAINMAST are (1) the number of trees that were explored during the tabu search and (2) the thresholds of density values in the map. Regarding the search space of tree structures, the current version explores 10 times more tree structures than the 2016 version. The density thresholds for the 2016 version were 100% and 50% of the author recommended contour level, while that for the 2017 version were 50% and 25%.

## 3. Results

We first summarize and discuss the models we generated for the Map Challenge. Then, we discuss different protocols newly applied to the targets. Finally, we show that counting consensus of top MAINMAST models can indicate confidence of local regions of a model.

### 3.1. Overview of the performance in the 2015/2016 E M Challenge

During the 2015/2016 Modeling Challenge, we made protein structure models for maps from all targets but two, T0003 (GroEL) and T0008 (70S Ribosome). We omitted these two targets because the density map of T0003 (EMD-6422) does not cover the reference PDB structure in some regions, and T0008 was too large to perform map segmentation. Since two different density maps were provided for T0006 (β-galactosidase) and T0007 (γ-secretase), in total we constructed models for eight maps from the six targets. Table 1 is the summary of the predicted models. For each of the map, top 10 models ranked by the Rosetta free energy were evaluated. Among the models we generated, we submitted models for only four targets, T0002, T0004, T0005, and T0007 (EMD-3061), to the Modeling Challenge organizers. Our models submitted to the Modeling Challenge and associated metadata have been archived at https://doi.org/10.5281/zenodo.1165999.

We did not submit models for the rest, because those models we generated for the remaining four maps (EMD-2842 from T0001, EMD-5995 and 2984 from T0006, and EMD-2677 from T0007) had a large root-mean square deviation (RMSD) to the author deposited models and we thought it was not worthwhile to submit the obviously low-quality models. Table 1 include both submitted and unsubmitted models.

**Fig. 2.**
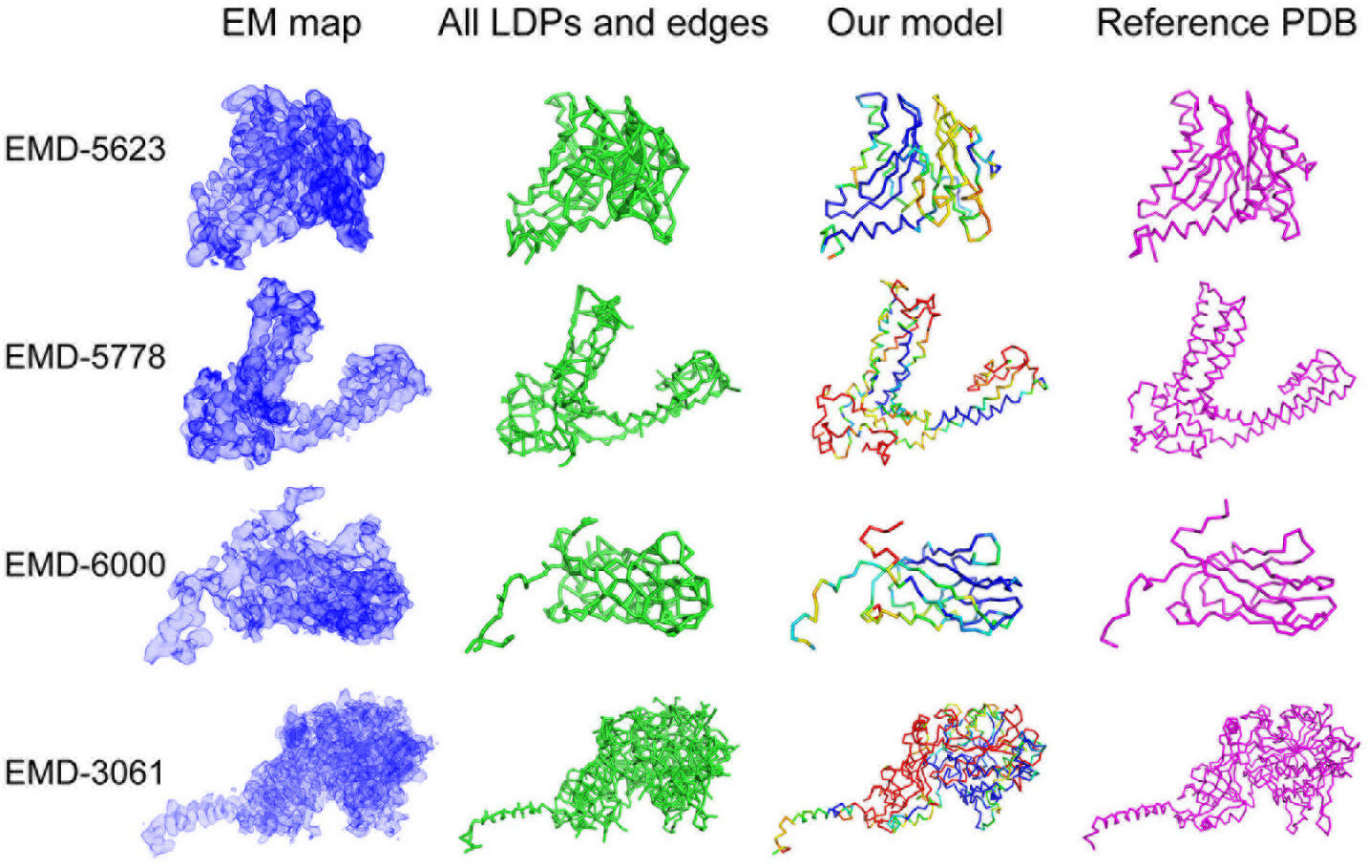
The best GDT-TS model among the top 10 scoring models generated by our modeling protocol for four target EM maps. Models were ranked by the Rosetta free energy in the refinement step. These models were submitted to the Model Challenge. The figure on the left (EM map) at each row is the segmented density map that was used as input. The author recommended contour level was used for visualization. The second column shows all LDPs and edges that were considered in the tabu search. The third column is the best GDT-TS model within the top 10 scoring models. The final column is the reference structure that were fitted to EM map by the authors. Our models are colored according to the deviation of Cα positions from the reference structure from blue (less than 1.0 Å) to red (over 8.0 Å).

**Fig. 3.**
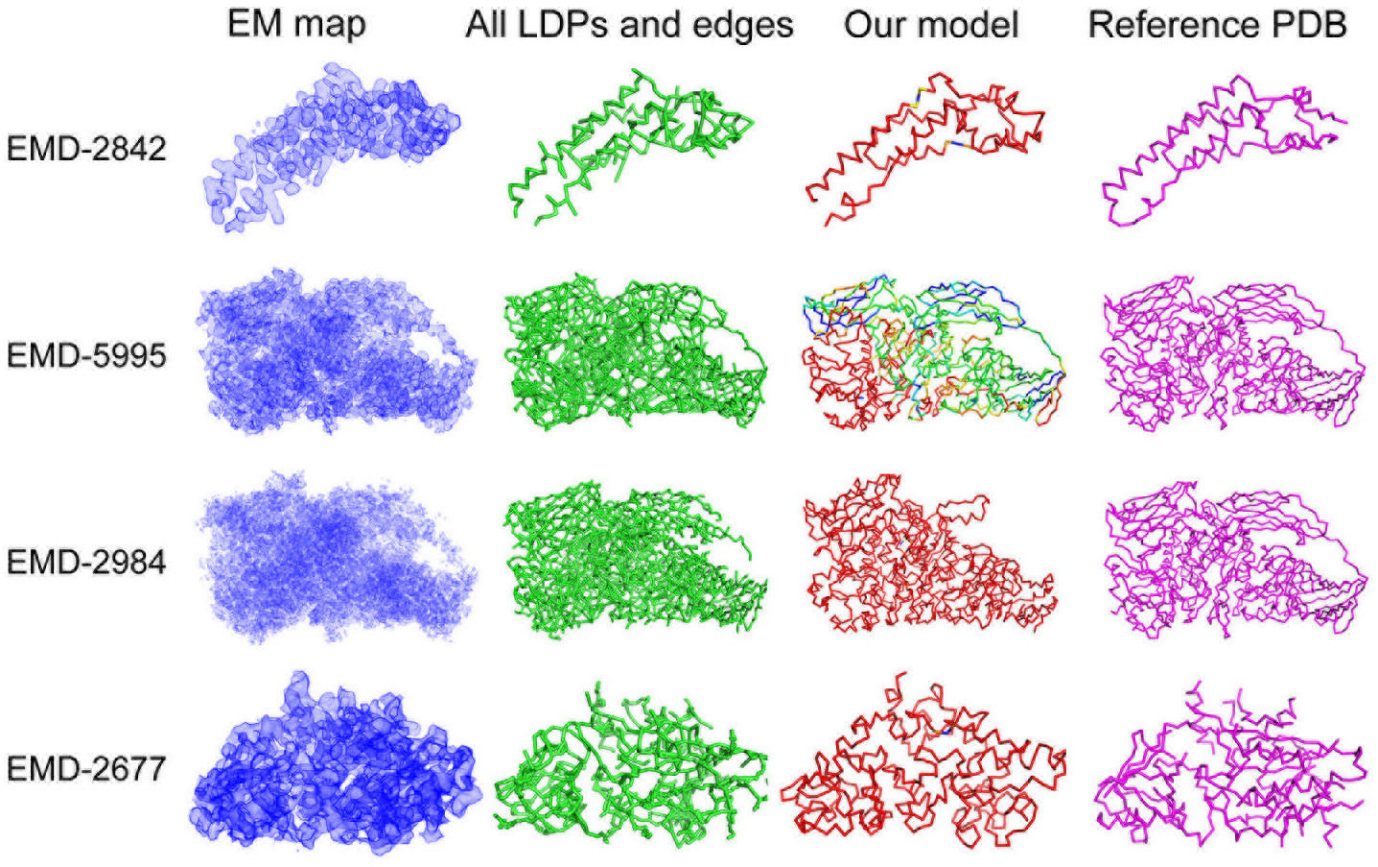
The best GDT-TS models among top 10 scoring models for the other four target maps (EMD-2842, 5995, 2984, and 2677). In these four targets, RMSD values of the top 10 model are worse than 10.0 Å. The color code of our models are the same as Fig. 2.

In Table 1, models were evaluated relative to the associated reference structure to each map. Five metrics were used: two sequence-dependent measures (RMSD and GDT-TS) and three sequence-independent measures (unlabeled RMSD, recall, and precision). An RMSD of a model is computed for Cα atoms. GDT-TS is an average percentage of Cα atoms in a model that are superimposed into the reference structure within four distance cutoffs, 1.0, 2.0, 4.0, and 8.0 Å (Zemla et al., 1999). GDT-TS ranges from 0 to 100 from the worst to the best score. These two metrics consider the accuracy of Cα atom positions and amino acid sequence assigments. An unlabeled RMSD computes deviation of Cα atoms of a model to the closest Cα atoms in the reference structure without considering the sequence matching. Recall is the fraction of Cα atoms in the reference structure which are closer than a threshold distance to any Cα atoms of a model. Precision is opposite, the fraction of Cα atoms in a model which are closer than a threshold distance to any Cα atoms in the reference structure. We used distance thresholds of 2.0 and 3.0 Å. Figs. 2 and 3 show the best model (in terms of GDT-TS) among the top 10 scoring models of the eight target EM maps. Fig. 2 are for target EM maps where the modeling was relatively successful, all with the best model among the top 10 top scoring models below an RMSD of 10.0 Å. Models for these maps were submitted to the Model Challenge. Fig. 3 are for the other cases, where the RMSD of the top 10 scoring models was all worse than 10.0 Å.

**Fig. 4.**
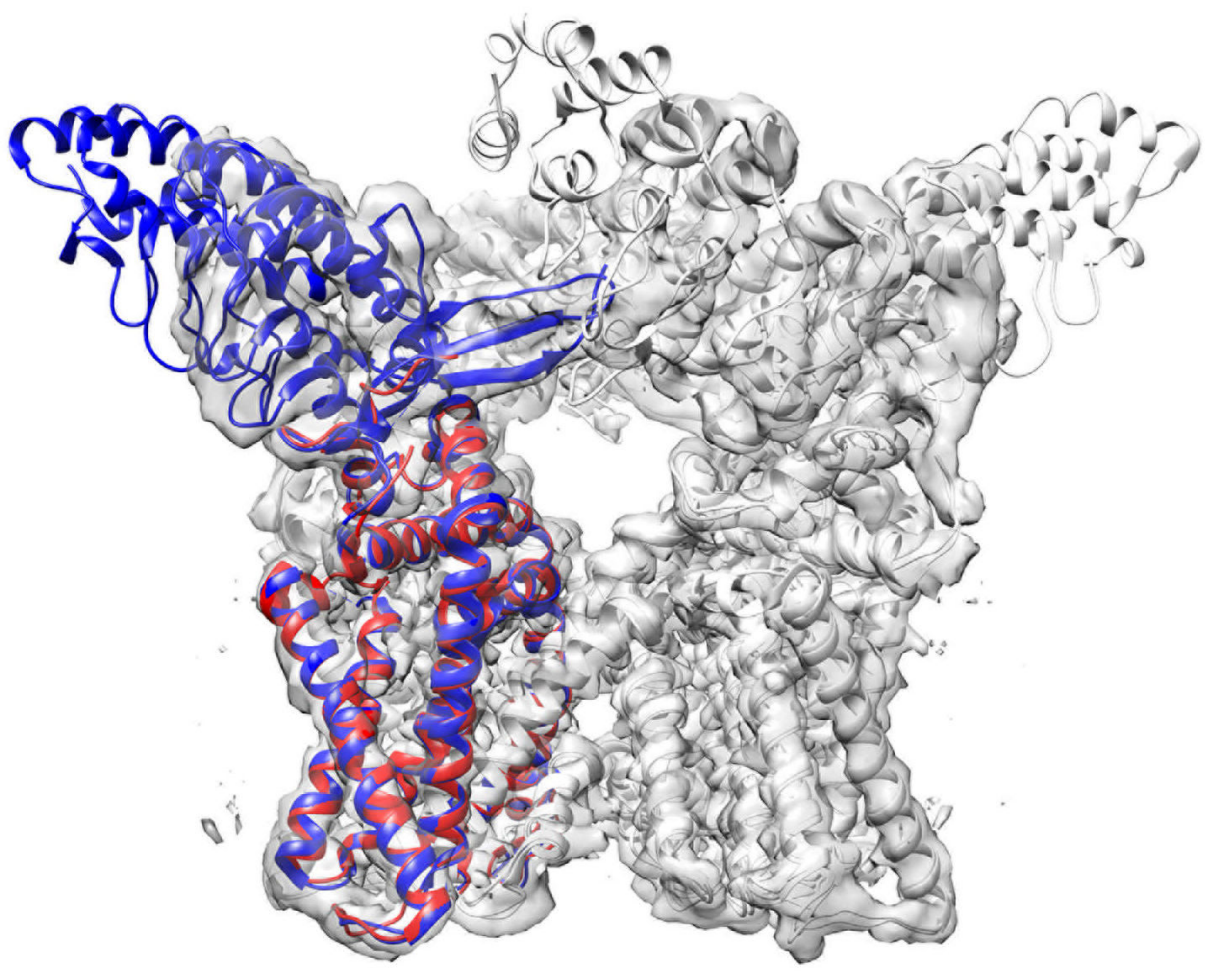
Superposition of two reference structures used for T0004 (EMD-5778). In addition to the reference structure, 3j5p-A (652 residues long including missing residues in the structure), which was specified by the Model Challenge organizers, we also used 3j9j-A (315 residue long) for assessing the models because we modelled structures using a segmented map of 3j9j-A. Thus, the length of our models is 315 residues, the same as 3j9j-A. 3j9j-A covers only the transmembrane domain of TrpV1 channel. The map of TrpV channel consists of four chains, and only chain A is colored in the figure (blue: 3j5p-A; red: 3j9j-A). The RMSD of the two structures at the overlapped region (310 residues) is 1.06 Å. The contour level shown is 7.0, which is the author-recommended contour value.

For the EM maps shown in Fig. 2, protein structures were modelled with an overall correct topology (main-chain conformation). Models of these maps have all an RMSD less than 10.0 Å to the reference structures (Table 1), and reflecting that, they all have a high recall and a precision with the singular exception of T0004. Except for T0004, the models correctly identified 93–99% of Cα atom positions in the reference structure (recall) and 96–99% of the Cα atom positions in the models are close enough to Cα atoms in the reference structure (precision) using a cutoff of 3.0 Å. In terms of unlabeled RMSD, all the models are within 2.2 Å. For the T0004 models, recall computed with 3j5p-A (the blue chain in Fig. 4) were particularly low. This is because we built models that only cover the transmembrane region of this channel protein using a segmented map based on 3j9j-A (the red chain in Fig. 4). We used 3j9j-A as the base of our modeling because 3j5p-A does not obviously fit into the density map of EMD-5778.

We now turn our attention to the graph structures of LDPs and edges provided in Fig. 2, which show all the connections between neighboring LDPs generated during the tabu search. Fig. 2 shows that α-helices are well captured and visible in the graphs. β- sheets, such as those in EMD-5623 and EMD-6000, tend to form a large number of connections in a graph, which in general makes tracing correct path difficult.

Fig. 3 show models for maps for which modelling were not very successful; all the top 10 scoring models of these maps have an RMSD over 10.0 Å. T0001 (EMD-2842) is an interesting case. As it is shown in the figure, the model identified main-chain positions accurately, however, the direction of the sequence and chain connections was inverted. Reflecting this, although the model has low GDT-TS, it has a small unlabeled RMSD and high recall and precision. EMD-5995 and EMD-2984 are for the same protein, β-galactosidase, which is the longest protein with 1022 residues among all the targets (The length of the other targets ranges from 149 to 665 residues). Examining the models of the two maps in Fig. 3, the model for the former has a topology and the chain direction (shown with chain colors) that are almost correct but with a small variation in the conformation of the N-terminal region (shown in blue). On the other hand, the model for EMD-2984 missed a β-sheet domain on the upper right corner of the figure and the chain direction is incorrect. The main reason of missing the β-sheet domain is that the local density of that region in this map is low, lower than EMD-5995 (indicated in the map density on the left column in Fig. 3) and because of that, MAINMAST could not see local dense points in that region. However, as also indicated by high recall and precision for this map (over 0.8) and a low unlabeled RMSD, most of the main-chain location were detected correctly. The model for the last density map, EMD-2677, obviously has an incorrect sequence direction and connections; however, many local structures were correctly captured.

### 3.2. Performance comparison with different settings of the modeling procedure

In this section, we compared the models generated in the Model Challenge with models newly built in different protocols. Table 2 compares Cα models built by two versions of MAINMAST, ones that were built in 2016 and new models generated for this work using the 2017 version. As mentioned in the Materials and Methods section, the 2017 version explores more tree structures and uses different contour levels of the density maps. Furthermore, three structure refinement tools were tested, Rosetta, which was used for generating and ranking models in the Model Challenge, as well as MDFF and xMDFF. xMDFF is originally developed for modeling structures for electron densities from X-ray crystallography. xMDFF has density map potentials of different resolutions, a potential for Cα atoms only, backbone atoms only, and for full atoms, which we ran sequentially before finishing with an energy minimization. The settings and the parameters used for running the three refinement methods are provided in Supplemental Note.

Models were evaluated in terms of GDT-TS in Table 2. Thus, a model with a larger value is closer to the reference structure. First, we compare the quality of Cα models by the two M AINM AST versions. On average, the 2016 version had a higher GDT-TS score of 19.3 as compared to 12.8 by the 2017 version when the 1st scoring models were considered while the 2017 version had a slightly higher score, 23.5, as compared to 23.2 by the 2016 version when top10 was considered. But overall their performance was similar. Comparing the three refinement protocols, Rosetta, MDFF, and xMDFF, we found again that their overall performance in terms of GDT-TS were very similar, although there are small difference for each target. For some targets we see that GDT-TS drastically increased after a refinement relative to the Cα model quality, e.g. from 3.6 to 27.3 for the 1st model by the 2017 version for T0007, EMD-3061. This substantial improvement occurred because the refinement protocol selected a different, better, model than what was selected by the threading score of M AINMAST.

**Fig. 5.**
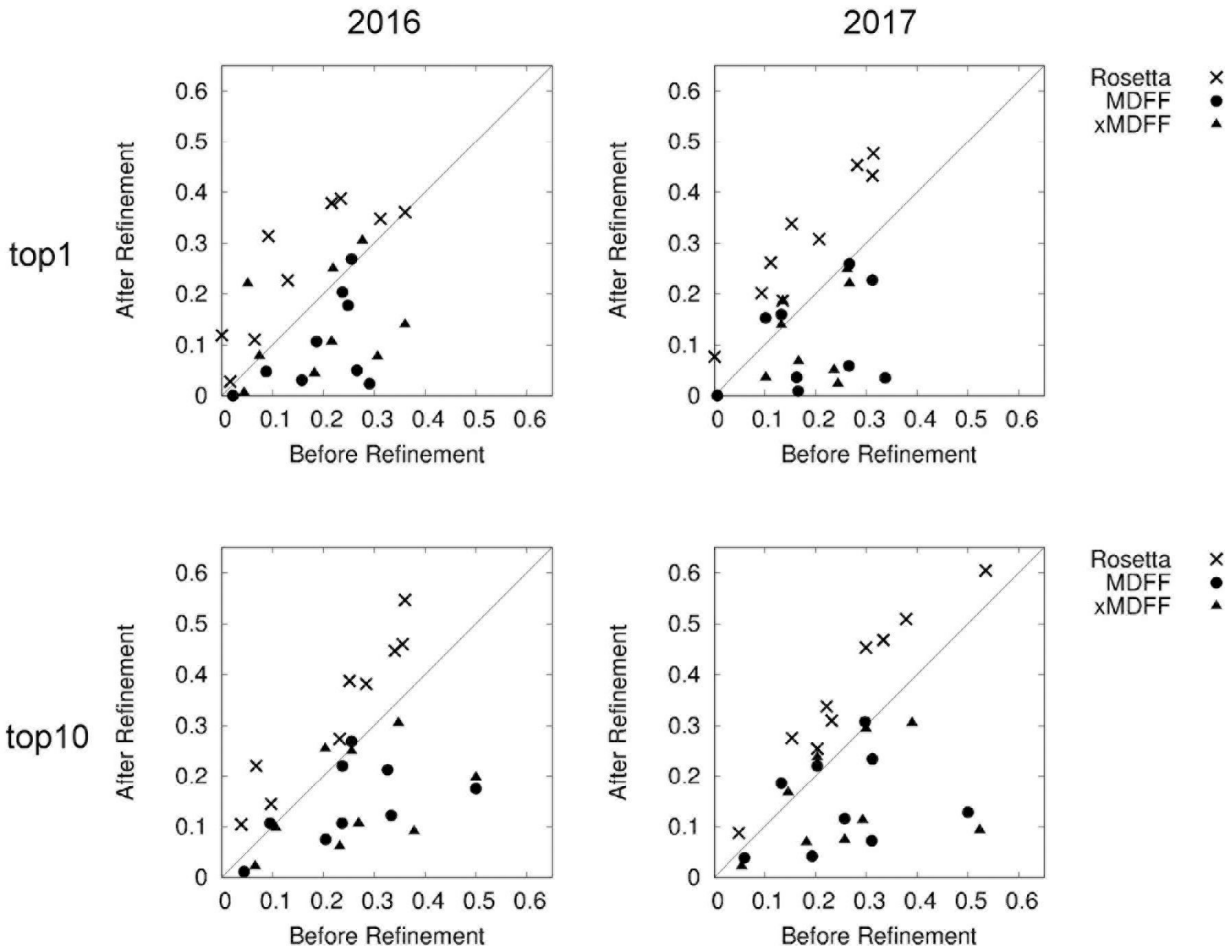
Comparison of the secondary structures before and after the refinement by Rosetta, MDFF, and xMDFF. The fraction of correctly modelled secondary structures were plotted before and after the refinement, which was applied to full atom models generated by PULCHRA. ×, Rosetta; ●, MDFF; and ▲, xMDFF. Refined models for the nine maps were selected by each refinement method (Top 1 and best among Top 10) and the changes of the correct secondary structure fraction to the models before and after the refinement method were plotted.

**Fig. 6.**
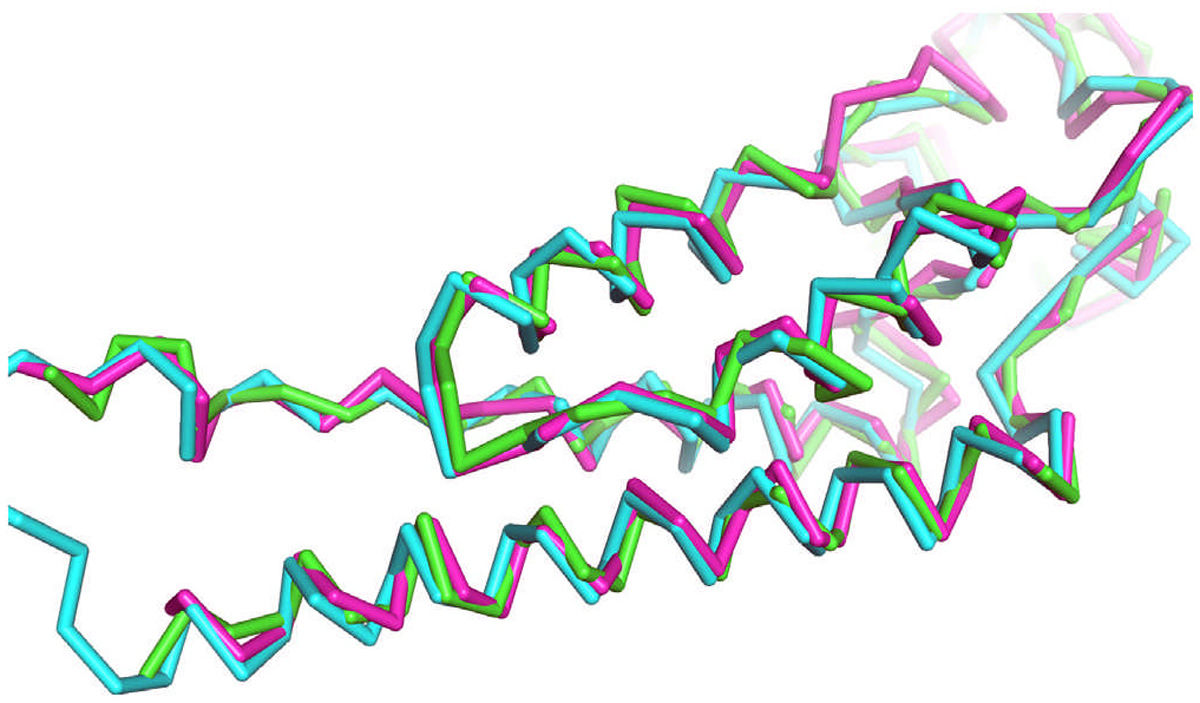
An example of changes made by MDFF. This is the top 10 MDFF model (i.e. the best RMSD model among top 10 scoring models ranked by MDFF) for the map EMD-2842. MAINMAST version 2016 was used. Cyan, the reference structure (PDB ID: 4udv); green, the PULCHRA model (i.e. before refinement); and magenta, after refinement by MDFF. Correct secondary structure fraction decreased from 0.5 to 0.17 by the MDFF refinement. Unlabeled RMSD was improved by MDFF from 1.48 Å to 1.46 Å.

In Fig. 5, we compared the fraction of correctly modelled secondary structures before and after the refinement by Rosetta, MDFF, and xMDFF, which were applied to full atom models built by PULCHRA. We used the DSSP program (Kabsch and Sander, 1983) to identify secondary structures in the models. The plots on the left column show results that used the MAINM AST version 2016 while on the right are results by the 2017 version. The top and the bottom rows are results for the best (top 1) scoring models and the best RMSD models among the top 10 scoring models ranked by each refinement protocol, respectively. The fraction of correctly modelled secondary structures detected by DSSP was not very high, all around 0.5 or lower. The plots show that Rosetta consistently improved secondary structures while MDFF and xMDFF decreased the fraction of correctly modelled secondary structures in most of the cases. Although MDFF and xMDFF deteriorated secondary structures according to the plots in Fig. 5, actual structural changes made were not large. Fig. 6 shows an example where MDFF decreased the fraction of correct secondary structures substantially from 0.50 (the PULCHRA model) to 0.17 (after refinement by MDFF). As can be seen in the figure, the Cα positions of the models did not change much before (green) and after (magenta) the MDFF refinement. The unlabeled Cα RMSD actually improved from 1.48 Å to 1.46 Å by the MDFF refinement despite of the decrease of the correct secondary structure fraction.

### 3.3. Confidence score of models

**Fig. 7.**
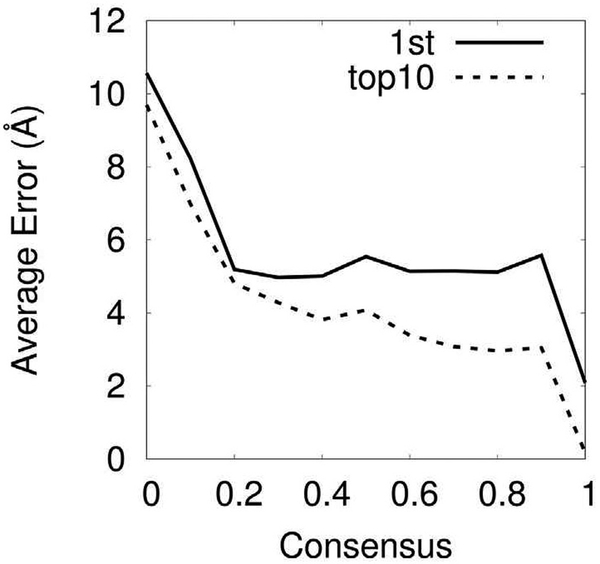
Average accuracy of residue positions relative to the degree of consensus among the top 100 scoring models for the five Modeling Challenge targets (EMD-3061, 5623, 5778, 5995, and 6000). We exclude other three targets (EMD-2842, 2984, 2677) due to their low GDT-TS scores of the top 10 models.

**Fig. 8.**
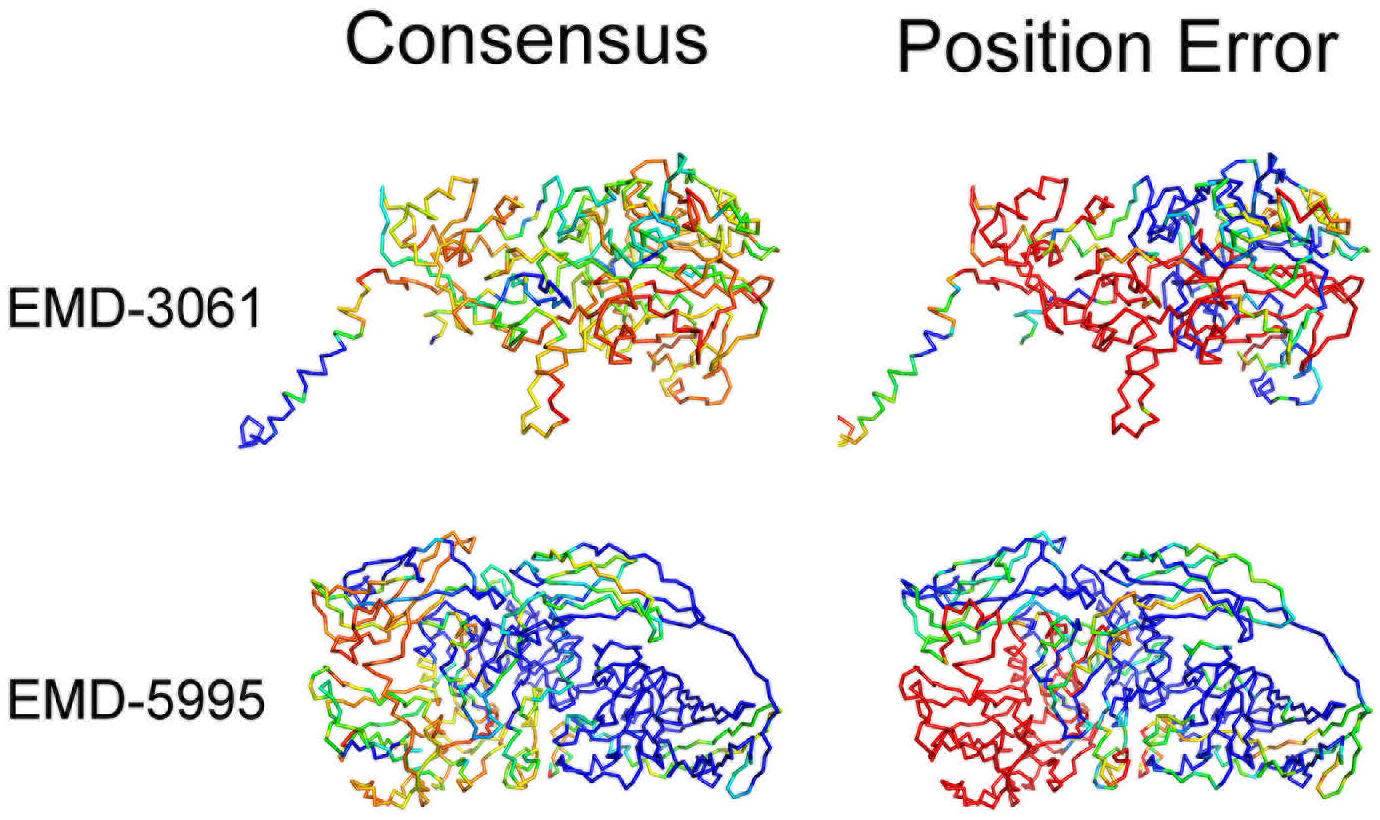
The best model among top 10 scoring models for EMD-3061 and EMD-5995 colored by the confidence (consensus) score. In the left column, the models are colored by the degree of consensus with red to blue for low to high degree of consensus. In the right column, the models are colored by the deviation of Cα positions from the reference structure with blue to red for small to large deviation.

A unique feature of MAINMAST is that it provides a confidence score for each Cα atom position of a model by considering consensus of top 100 scoring models ranked by the threading score (i.e. the sequence to main-chain mapping score). To compute the confidence score of a model, for each Cα atom position the number of models that have the atom within 3.5 Å among top 100 scoring models are counted. Intuitively, a residue position in a model is expected to be more accurate if it is a strong consensus among alternative high-scoring models. Fig. 7 shows that this is the case in general. The average error of Cα positions decreased in general as the consensus degree increases. In particular, the error showed a sharp drop when over 80% (0.8 in the plot) of models agree to atom positions. Fig. 8 shows two examples of the models from EMD-3061 and EMD-5995. In both cases, the large position error (positions in red on the right panels) corresponds well with the less consensus regions (blue in the left panels).

## 4. Discussion

The Model Challenge provided a valuable opportunity for us to evaluate the performance of our modeling protocol using M AINMAST. Overall we are pleased to see that M AINM AST was able to trace main-chains of proteins in the density maps well, capturing most of the main-chain positions of the target proteins and built almost correct topology for most of the maps. Even in the cases that models did not have good values in sequence-dependent numerical metrics i.e. GDT-TS and RMSD, we observed that the models have correct local fragments of protein structures and the graph structures of LDPs (Fig. 3) capture the characteristics of the conformation of the proteins. Thus, protein models as well as the graph structures will be able to aid biologists in a model building procedure. Many alternative models and the consensus score computed from them would be also valuable information for manual model building.

On the other hand, major errors were caused by incorrect connections of identified fragments. We also observed an interesting error that a protein sequence was mapped on the opposite direction to a correctly identified main-chain trace (Fig. 3, EMD-2842). Figs. 2 and 3 showed that building a perfect model for large proteins is difficult. We observed for EMD-2984 (Fig. 3) that a substantial error occurred at a map region with a lower density, which is not trivial to correct because the map itself does not have sufficient structure information in that region.

A potential direction for further improvement of the protocol would be to incorporate information of known protein structures from PDB. MAINMAST simply connects locally dense points in an EM map and currently does not use any information from known protein structures. In one sense this is a strength; however, it is reasonable to expect that knowledge of existing protein structures, global or local, would be helpful in several situations, including for improvement of detailed stereochemistry of structures (e.g. constructing regular α helices and β strands) and chain modeling of low-density map regions. As investigated in Table 2, small tweaks of parameters and using different refinement programs did not make much difference in the accuracy; thus, some drastic implementation would be needed to achieve meaningful improvement.

Finally, we would like to mention the difficulty of evaluating models from EM maps. Since maps are not determined at a X-ray crystal­lography resolution (i.e. ~ 1.5 Å), strictly speaking maps do not have sufficient information to determine precise atom positions of proteins. In this Model Challenge, the organizers as well as we compared models with author-deposited structures as reference and computed numerical metrics including RMSD, but it is noted that with a map in this resolution range (~ 3 Å) a fitted structure can easily move 1 Å or more in the map by applying structure refinement protocol (Monroe et al., 2017). Thus, better ways to assess accuracy of models from EM map would remain as an important topic of discussion by the community.

## 5. Conclusion

We participated in the 2015/2016 Model Challenge by building models using MAINMAST. The results show that our protocol was able to build models of the correct conformation for a few maps while local structures but not the sequence mapping were correct for the other maps.

## Supplementary Material

1

## Figures and Tables

**Table 1:** Summary of the models for 2015/2016 Modeling Challenge target maps.

Target	EMDB-ID^a^	Res. (Å)	PDB^b^	Model^c^	RMSD (A)^d^	GDT-TS^e^	Unlabeled RMSD^f^	Recall d < 2/3Å^g^	Precision d < 2/3Å^h^
T0001	2842	3.3	4udv-A	1st	11.7	17.0	1.6	0.83/0.97	0.84/0.96
Tabacco Mosaic Virus				top10	11.4	19.6	1.6	0.84/0.97	0.85/0.98
T0002	5623	3.3	3j9i-A	1st	5.1	46.2	1.6	0.79/0.97	0.80/0.98
T20S Proteasome				top10	3.7	58.8	1.5	0.85/0.99	0.86/0.98
T0004	5778	3.3	3j5p-A (3j9j-A)	1st	9.2 (9.1)	15.6 (30.0)	2.1 (2.2)	0.34/0.48 (0.68/0.93)	0.64/0.87 (0.68/0.88)
TrpV1 Channel				top10	8.5 (8.3)	18.6 (36.0)	2.0 (2.1)	0.36/0.50 (0.68/0.94)	0.69/0.90 (0.68/0.89)
T0005	6000	3.8	3j71-A	1st	3.4	60.7	1.6	0.79/0.99	0.79/0.97
Bromo Mosaic Virus				top10	3.4	60.7	1.6	0.81/0.99	0.81/0.99
T0006	5995	3.2	3j7h-A	1st	12.4	50.6	1.6	0.80/0.93	0.80/0.96
β-Galactosidase				top10	11.7	52.7	1.5	0.84/0.94	0.85/0.96
T0006	2984	2.2	5a1a-A	1st	29.9	2.6	2.1	0.64/0.81	0.65/0.87
β-Galactosidase				top10	27.7	3.6	2.0	0.66/0.82	0.67/0.90
T0007	2677	4.5	4upc-A	1st	27.2	4.5	2.7	0.38/0.67	0.39/0.74
γ-Secretase				top10	23.9	5.6	2.6	0.38/0.70	0.39/0.75
T0007	3061	3.4	5a63-A	1st	11.4	17.7	1.7	0.77/0.96	0.77/0.96
γ-Secretase				top10	8.8	37.3	1.6	0.82/0.96	0.82/0.96
Average				1st	13.3	27.2	1.9	0.67/0.86	0.71/0.91
				Top10	11.9	32.5	1.8	0.70/0.87	0.73/0.92

a Density maps whose models were submitted to the official assessment are underlined.

b The reference PDB structure, against which models were compared. We only modeled the A chain of each complex. For EMD-5778, the map was segmented based on 3j9j-A and models were assessed with two reference structures, 3j5p-A, which was provided at the Map Challenge website, and 3j9j-A. See text for details. Residue numbers in MAINMAST models were renumbered based on the reference PDB structures when assessed.

c 1st, the top scoring model; top10, the best GDT-TS model among top 10 scoring models. All models were ranked by the Rosetta Free Energy.

d The RMSD of Cα atoms modelled by MAINMAST and the reference structure.

e Global distance test total score. The value ranges from 0 to 100 with 100 as the best score.

f The RMSD between nearest Cα atoms of MAINMAST and reference structure.

g The fraction of Cα atoms in the reference structure which are closer than a threshold distance (2.0 or 3.0 Å) to any Cα atoms in the model.

h The fraction of Cα atoms in the model which are closer than a threshold distance (2.0 or 3.0 Å) to any Cα atoms in the reference structure.

**Table 2:** Comparison of performance of two versions of MAINMAST & three refinement methods.

Target	EMDB-ID	PDB	Model	2016ver^a^				2017ver^b^			
				Cα Model^c^	Rosetta^d^	MDFF^e^	xMDFF^f^	Cα Model^c^	Rosetta^d^	MDFF^e^	xMDFF^f^
T0001	2842	4udv-A	1st	19.9	17.0	12.9	16.3	14.4	14.2	14.4	12.3
Tabacco Mosaic Virus			top10	19.9	19.6	17.2	16.8	14.4	18.8	16.7	19.3
T0002	5623	3j9i-A	1st	47.2	46.2	44.2	47.2	25.8	63.4	63.8	60.3
T20S Proteasome			top10	49.8	58.8	55.6	54.5	56.6	63.4	63.8	62.8
T0004	5778	3j5p-A	1st	14.5	15.6	15.0	18.9	14.9	5.1	15.1	12.0
TrpV1 Channel			top10	17.9	18.6	21.4	18.9	19.5	18.7	20.7	19.6
T0004	5778	3j9j-A	1st	27.4	30.0	28.8	36.4	27.7	9.3	28.2	22.7
TrpV1			top10	34.0	36.0	41.0	36.4	36.8	36.1	39.5	39.5
Channel											
T0005	60BA	3j71-A	1st	10.6	60.7	65.9	63.9	15.8	62.8	62.4	60.1
Bromo Mosaic Virus			top10	22.3	60.7	65.9	63.9	15.8	66.3	66.4	67.8
T0006	5995	3j7h-A	1st	42.0	50.6	50.4	52.0	3.9	56.1	57.5	55.9
β-Galactosidase			top10	51.5	52.7	52.5	53.0	54.9	56.1	58.1	55.9
T0006	2984	5a1a-A	1st	2.9	2.6	2.5	2.7	2.7	19.7	16.6	16.1
β-Galactosidase			top10	3.3	3.6	3.0	2.7	2.7	25.4	18.2	16.1
T0007	2677	4upc-A	1st	5.1	4.5	4.3	4.5	6.7	4.8	5.8	5.3
γ-Secretase			top10	5.4	5.6	5.4	5.0	6.7	6.5	5.9	6.0
T0007	3061	5a63-A	1st	3.8	17.7	27.2	17.4	3.6	27.3	22.4	16.9
γ-Secretase			top10	4.5	37.3	36.3	36.3	4.2	29.4	22.4	21.0
Average			1st	19.3	27.2	27.9	28.8	12.8	29.2	31.8	29.0
			Top10	23.2	32.5	33.1	31.9	23.5	35.6	34.6	33.9

Models are evaluated in terms of the GDT-TS score (the larger the better, the maximum score is 100). For each raw, the best (largest) value is indicated in bold.

a Models from the MAINMAST 2016 version that was used for the 2015/2016 Modeling Challenge.

b Models from the latest version of MAINMAST.

c Cα models from MAINMAST before refinement.

d Refined models by Rosetta refinement protocol. Please see the Methods section for details.

e Refined models by MDFF.

f Refined models by xMDFF.

## References

[R1] Afonine PV, Poon BK, Read RJ, Sobolev OJ, Terwilliger TC, Urzhumtsev A, Adams PD, 2018. Real-Space Refinement in Phenix for Cryo-EM and Crystallography. bioRxiv 10.1107/S2059798318006551 PMC6096492 29872004

[R2] Baker ML, Ju T, Chiu W, 2007. Identification of secondary structure elements in intermediate-resolution density maps. Structure 15, 7–19. 17223528 10.1016/j.str.2006.11.008 PMC1810566

[R3] Baker ML, Baker MR, Hryc CF, Ju T, Chiu W., 2012a. Gorgon and pathwalking: macromolecular modeling tools for subnanometer resolution density maps. Biopolymers 97, 6 55–668. 10.1002/bip.22065 PMC3899894 22696403

[R4] Baker MR, Rees I, Ludtke SJ, Chiu W., Baker ML, 2012b. Constructing and validating initial Calpha models from subnanometer resolution density maps with pathwalking. Structure 20, 4 50–463. 10.1016/j.str.2012.01.008 PMC3307788 22405004

[R5] Chen H, Kihara D, 2011. Effect of using suboptimal alignments in template-based protein structure prediction. Proteins 79, 3 15–334. 10.1002/prot.22885 PMC3058269 21058297

[R6] Chen M, Baldwin PR, Ludtke SJ, Baker ML, 2016. De Novo modeling in cryo-EM density maps with Pathwalking. J. Struct. Biol 196, 2 89–298. 10.1016/j.jsb.2016.06.004 PMC5118137 27436409

[R7] DiMaio F, Tyka MD, Baker ML, Chiu W, Baker D, 2009. Refinement of protein structures into low-resolution density maps using rosetta. J. Mol. Biol 392, 181–190. 19596339 10.1016/j.jmb.2009.07.008 PMC3899897

[R8] DiMaio F, Song Y, Li X, Brunner MJ, Xu C, Conticello V, Egelman E, Marlovits TC, Cheng Y, Baker D, 2015. Atomic-accuracy models from 4.5-A cryo-electron microscopy data with density-guided iterative local refinement. Nat. Methods 12, 3 61–365. 25707030 10.1038/nmeth.3286 PMC4382417

[R9] Esquivel-Rodriguez J, Kihara D, 2012. Fitting multimeric protein complexes into electron microscopy maps using 3D zernike descriptors. J. Phys. Chem. B 116, 6854–6861. 22417139 10.1021/jp212612t PMC3376205

[R10] Esquivel-Rodriguez J, Kihara D, 2013. Computational methods for constructing protein structure models from 3D electron microscopy maps. J. Struct. Biol 184, 93–102. 23796504 10.1016/j.jsb.2013.06.008 PMC3795986

[R11] Frank J, 2017. Advances in the field of single-particle cryo-electron microscopy over the last decade. Nat Protoc 12, 2 09–212. 10.1038/nprot.2017.004 PMC5479931 28055037

[R12] Frenz B, W alls AC, Egelman EH, Veesler D, DiM aio F, 2017. RosettaES: a sampling strategy enabling automated interpretation of difficult cryo-EM maps. Nat. Methods 14, 7 97–800. 10.1038/nmeth.4340 PMC6009829 28628127

[R13] Fukunaga K, Hostetler LD, 1975. Estimation of gradient of a density-function, with applications in pattern-recognition. IEEE Trans. Inf. Theory 21, 32–40.

[R14] Glover F, 1986. Future paths for integer programming and links to artificial-intelligence. Comput. Oper. Res 13, 533–549.

[R15] Hohn M, Tang G, Goodyear G, Baldwin PR, Huang Z, Penczek PA, Yang C, Glaeser RM, Adams PD, Ludtke SJ, 2007. SPARX, a new environment for Cryo-EM image processing. J. Struct. Biol 157, 47–55. 16931051 10.1016/j.jsb.2006.07.003

[R16] Jiang W., Baker ML, Ludtke SJ, Chiu W, 2001. Bridging the information gap: computational tools for intermediate resolution structure interpretation. J. Mol. Biol 308, 1033–1044. 11352589 10.1006/jmbi.2001.4633

[R17] Kabsch W., C. Sander, 1983. Dictionary of protein secondary structure: pattern recognition of hydrogen-bonded and geometrical features. Biopolym ers 22, 2577. 10.1002/bip.360221211 6667333

[R18] Kirmizialtin S, Loerke J, Behrmann E, Spahn CM, Sanbonmatsu KY, 2015. Using molecular simulation to model high-resolution cryo-EM reconstructions. Methods Enzym 558, 497–514. 10.1016/bs.mie.2015.02.011 26068751

[R19] Lopez-Blanco JR, Chacon P, 2013. iMODFIT: efficient and robust flexible fitting based on vibrational analysis in internal coordinates. J. Struct. Biol 184, 261–270. 23999189 10.1016/j.jsb.2013.08.010

[R20] Ludtke SJ, Lawson CL, Kleywegt GJ, Berman H, Chiu W, 2012. The 2010 cryo-EM modeling challenge. Biopolymers 97, 651–654. 22696402 10.1002/bip.22081

[R21] McGreevy R, Teo I, Singharoy A, Schulten K, 2016. Advances in the molecular dynamics flexible fitting method for cryo-EM modeling. Methods 100, 50–60. 26804562 10.1016/j.ymeth.2016.01.009 PMC4848153

[R22] McGreevy R, Singharoy A, Li Q, Zhang J, Xu D, Perozo E, Schulten K, 2014. xMDFF: molecular dynamics flexible fitting of low-resolution X-ray structures. Acta Crystallogr. D Biol. Crystallogr 70, 2344–2355. 10.1107/S1399004714013856 PMC4157446 25195748

[R23] Miyashita O, Kobayashi C, Mori T, Sugita Y, Tama F, 2017. Flexible fitting to cryo-EM density map using ensemble molecular dynamics simulations. J. Comput. Chem 38, 1447–1461. 28370077 10.1002/jcc.24785

[R24] Monroe L, Terashi G, Kihara D, 2017. Variability of protein structure models from electron microscopy. Structure 25 (592–602), e592. 10.1016/j.str.2017.02.004 PMC5382112 28262392

[R25] Patwardhan A, 2017. Trends in the electron microscopy data bank (EMDB). Acta Crystallogr. D Biol. Crystallogr 73, 503–508. 10.1107/S2059798317004181 PMC5458492 28580912

[R26] Punjani A, Rubinstein JL, Fleet DJ, Brubaker MA, 2017. cryoSPARC: algorithm s for rapid unsupervised cryo-EM structure determination. Nat. Methods 14, 290–296. 28165473 10.1038/nmeth.4169

[R27] Rotkiewicz P, Skolnick J, 2008. Fast procedure for reconstruction of full-atom protein models from reduced representations. J. Comput. Chem 29, 1460–1465. 18196502 10.1002/jcc.20906 PMC2692024

[R28] Scheres SH, 2012. RELION: implementation of a Bayesian approach to cryo-EM structure determination. J. Struct. Biol 180, 519–530. 23000701 10.1016/j.jsb.2012.09.006 PMC3690530

[R29] Singharoy A, Teo I, McGreevy R, Stone JE, Zhao J, Schulten K, 2016. Molecular dynamics-based refinement and validation for sub-5 A cryo-electron microscopy maps. eLife 5, e16105. 27383269 10.7554/eLife.16105 PMC4990421

[R30] Skolnick J, Kihara D, 2001. Defrosting the frozen approximation: PROSPECTOR-a new approach to threading. Proteins 42, 319–331. 11151004

[R31] Tang G, Peng L, Baldwin PR, Mann DS, Jiang W, Rees I, Ludtke SJ, 2007. EM AN2: an extensible image processing suite for electron microscopy. J. Struct. Biol 157, 38–46. 16859925 10.1016/j.jsb.2006.05.009

[R32] Terashi G, Kihara D, 2018. De novo main-chain modeling for EM maps using MAINMAST. Nat. Commun 9,1618. 29691408 10.1038/s41467-018-04053-7 PMC5915429

[R33] Terwilliger TC, Grosse-Kunstleve RW, Afonine PV, M oriarty NW., Zwart PH, Hung LW, Read RJ, Adams PD, 2008. Iterative model building, structure refinement and density modification with the PHENIX AutoBuild wizard. Acta Crystallogr. D Biol. Crystallogr 64, 61–69. 10.1107/S090744490705024X PMC2394820 18094468

[R34] Trabuco LG, Villa E, Mitra K, Frank J, Schulten K, 2008. Flexible fitting of atomic structures into electron microscopy maps using molecular dynamics. Structure 16,6 73–683. 10.1016/j.str.2008.03.005 PMC2430731 18462672

[R35] Velankar S, van Ginkel G, Alhroub Y, Battle GM, Berrisford JM, Conroy MJ, Dana JM, Gore SP, Gutmanas A, Haslam P, Hendrickx PM, Lagerstedt I, Mir S, Fernandez Montecelo MA, Mukhopadhyay A, Oldfield TJ, Patwardhan A, Sanz-Garcia E, Sen S, Slowley RA, Wainwright ME, Deshpande MS, Iudin A, Sahni G, Salavert Torres J, Hirshberg M, Mak L, Nadzirin N, Armstrong DR, Clark AR, Smart OS, Korir PK, Kleywegt GJ, 2016. PDBe: improved accessibility of macromolecular structure data from PDB and EMDB. Nucl. Acids Res. 44, D385–395. 26476444 10.1093/nar/gkv1047 PMC4702783

[R36] Wang RY, Kudryashev M, Li X, Egelman EH, Basler M, Cheng Y, Baker D, DiMaio F, 2015. De novo protein structure determination from near-atomic-re solution cryo-EM maps. Nat. Methods 12, 335–338. 25707029 10.1038/nmeth.3287 PMC4435692

[R37] Woetzel N, Lindert S, Stewart PL, Meiler J, 2011. BCL::EM-Fit: rigid body fitting of atomic structures into density maps using geometric hashing and real space refinement. J. Struct. Biol 175, 264–276. 21565271 10.1016/j.jsb.2011.04.016 PMC3150432

[R38] Wriggers W, Birmanns S, 2001. Using situs for flexible and rigid-body fitting of multiresolution single-molecule data. J. Struct. Biol 133, 193–202. 11472090 10.1006/jsbi.2000.4350

[R39] Zemla A, Venclovas C, Moult J, Fidelis K, 1999. Processing and analysis of CASP3 protein structure predictions. Proteins (Suppl. 3), 22–29. 10526349 10.1002/(sici)1097-0134(1999)37:3+<22::aid-prot5>3.3.co;2-n

