# Supplementary material for: De novo main-chain modeling with MAINMAST in 2015/2016 EM Model Challenge
Source: J Struct Biol. Author manuscript; Available in PMC 2018 Nov 1. (PMC6179447; doi:10.1016/j.jsb.2018.07.013)
Supplement: 1 [file NIHMS991310-supplement-1.pdf]

Supplementary Infomration for

De novo main-chain modeling with MAINMAST in 2015/2016 EM Model Challenge

Genki Terashi[1,] and Daisuke Kihara[1,2,*]

[1]Department of Biological Sciences, Purdue University, West Lafayette, IN 47907, USA

[2]Department of Computer Science, Purdue University, West Lafayette, IN 47907, USA

* Contact: dkihara@purdue.edu

Supplementary Note 1. Settings and parameters used for running Rosetta, MDFF, and xMDFF.

Running Rosetta refinement protocol

Rosetta ver. 3.5 (rosetta_bin_linux_2015.19.57819) was used. We followed the tutorial released on http://dimaiolab.ipd.uw.edu/software/. Almost all the parameters used were as described in the tutorial:

```
$ROSETTA3/source/bin/rosetta_scripts.linuxgccrelease ¥
 -database $ROSETTA3/database/ ¥
 -in::file::s inp.pdb ¥
 -parser::protocol cycles.xml ¥
 -ignore_unrecognized_res ¥
 -edensity::mapreso $resolution ¥
 -edensity::cryoem_scatterers ¥
 -crystal_refine ¥
 -out::suffix _relax ¥
 -default_max_cycles 2000
```

Running MDFF

We followed the tutorial released on
http://www.ks.uiuc.edu/Training/Tutorials/science/mdff/tutorial_mdff-html/node4.html

1. Preparing files that were used in MDFF

In the *VMD Tk Console*, we executed the following commands:

```
package require mdff
package require cispeptide
package require chirality
package require ssrestraints
mdff griddx -i map.mrc -o map.dx
mol new target_autopsf.psf
mol addfile target_autopsf.pdb
```

```
mdff gridpdb -psf target_autopsf.psf -pdb target_autopsf.pdb -o
target-grid.pdb
cispeptide restrain -o target-cispeptide.txt
chirality restrain -o target-chirality.txt
```

2. Preparing NAMD configuration files for MDFF simulation and minimization

For MDFF simulation, we prepared the following NAMD configuration file:

target_N.namd

```
set PSFFILE target_autopsf.psf
set PDBFILE target_autopsf.pdb
set GRIDPDB target-grid.pdb
set GBISON 0
set DIEL 80
set SCALING_1_4 1.0
set ITEMP 300
set FTEMP 300
set GRIDFILE map.dx
set GSCALE 0.5
set EXTRAB {target-cispeptide.txt target-chirality.txt}
set CONSPDB 0
set FIXPDB  0
set GRIDON 1
set OUTPUTNAME target-N
set TS 1000000
set MS 10000
set MARGIN 0
structure $PSFFILE
coordinates $PDBFILE
paraTypeCharmm on
parameters par_all36_prot.prm

if {[info exists INPUTNAME]} {
```

```
  BinVelocities $INPUTNAME.restart.vel
  BinCoordinates $INPUTNAME.restart.coor
} else {
  temperature $ITEMP
}
source mdff_template.namd
```

For minimization, we prepared the following NAMD configuration file.

target_Nmini.namd

```
set PSFFILE target_autopsf.psf
set PDBFILE target_autopsf.pdb
set GRIDPDB target-grid.pdb
set GBISON 0
set DIEL 80
set SCALING_1_4 1.0
set ITEMP 300
set FTEMP 300
set GRIDFILE map.dx
set GSCALE 0.5
set EXTRAB {target-cispeptide.txt target-chirality.txt}
set CONSPDB 0
set FIXPDB  0
set GRIDON 1

set INPUTNAME target-N
set OUTPUTNAME target-Nmini
set TS 0
set MS 10000
set MARGIN 0
structure $PSFFILE
coordinates $PDBFILE

paraTypeCharmm on
```

```
parameters par_all36_prot.prm

if {[info exists INPUTNAME]} {
  BinVelocities $INPUTNAME.restart.vel
  BinCoordinates $INPUTNAME.restart.coor
} else {
  temperature $ITEMP
}
source mdff_template.namd
```

3. Executing MDFF simulation

```
#MDFF simulation
./charmrun ++local +p24 namd2 target_N.namd
#Minimization
./charmrun ++local +p24 namd2 target_Nmini.namd
```

Running xMDFF

We followed the tutorial released on http://www.ks.uiuc.edu/Training/Tutorials/science/mdff/tutorial_mdff-html/node8.html However, we could not use the reflection data for Modeling Challenge 2015/2016. Therefore, we exclude the part where the file containing the reflection data is required in the xMDFF protocol.

1. Preparing files.

The file preparation steps of xMDFF are same with the MDFF simulation. In the VMD Tk Console, we executed the following commands additionally:

```
mdff gridpdb -psf target_autopsf.psf -pdb target_autopsf.pdb -o
target-gridca.pdb -seltext "name CA”
mdff gridpdb -psf target_autopsf.psf -pdb target_autopsf.pdb -o
target-gridbb.pdb -seltext "backbone"
```

```
mdff gridpdb -psf target_autopsf.psf -pdb target_autopsf.pdb -o
target-grid.pdb
```

The three PDB files (target-gridca.pdb, target-gridbb.pdb and target-grid.pdb) are corresponding to the density map potential on C-alpha atoms, backbone atoms and all atoms, respectively.

2. Preparing NAMD configuration files

We prepared three NAMD configuration files, target_Xca.namd, target_Xbb.namd and target_Xall.namd.

target_Xca.namd

```
#Using Density Map Potential of C-alpha atom positions
set PSFFILE target_autopsf.psf
set PDBFILE target_autopsf.pdb
set GRIDPDB target-gridca.pdb
set GBISON 0
set DIEL 80
set SCALING_1_4 1.0
set ITEMP 300
set FTEMP 300
set GRIDFILE map.dx
set GSCALE 0.5
set EXTRAB { target-cispeptide.txt target-chirality.txt}
set CONSPDB 0
set FIXPDB  0
set GRIDON 1
set OUTPUTNAME target-Xca
set TS 1000000
set MS 10000
set MARGIN 0

structure $PSFFILE
```

```
coordinates $PDBFILE

paraTypeCharmm on
parameters par_all36_prot.prm

if {[info exists INPUTNAME]} {
  BinVelocities $INPUTNAME.restart.vel
  BinCoordinates $INPUTNAME.restart.coor
} else {
  temperature $ITEMP
}
source mdff_template.namd
```

target_Xbb.namd

```
#Using Density Map Potential of backbone atom positions
set PSFFILE target_autopsf.psf
set PDBFILE target_autopsf.pdb
set GRIDPDB target-gridbb.pdb
set GBISON 0
set DIEL 80
set SCALING_1_4 1.0
set ITEMP 300
set FTEMP 300
set GRIDFILE map.dx
set GSCALE 0.5
set EXTRAB {target-cispeptide.txt target-chirality.txt}
set CONSPDB 0
set FIXPDB  0
set GRIDON 1
set INPUTNAME target-Xca
set OUTPUTNAME target-Xbb
set TS 1000000
set MS 10000
```

```
set MARGIN 0
structure $PSFFILE
coordinates $PDBFILE

paraTypeCharmm on
parameters par_all36_prot.prm

if {[info exists INPUTNAME]} {
  BinVelocities $INPUTNAME.restart.vel
  BinCoordinates $INPUTNAME.restart.coor
} else {
  temperature ¥$ITEMP
}
source mdff_template.namd
```

target_Xall.namd

```
set PSFFILE target_autopsf.psf
set PDBFILE target_autopsf.pdb
set GRIDPDB target-grid.pdb
set GBISON 0
set DIEL 80
set SCALING_1_4 1.0
set ITEMP 300
set FTEMP 300
set GRIDFILE map.dx
set GSCALE 0.5
set EXTRAB { target-cispeptide.txt target-chirality.txt}
set CONSPDB 0
set FIXPDB  0
set GRIDON 1
set INPUTNAME target-Xbb
set OUTPUTNAME target-Xall
set TS 1000000
```

```
set MS 10000
set MARGIN 0
structure $PSFFILE
coordinates $PDBFILE

paraTypeCharmm on
parameters par_all36_prot.prm

if {[info exists INPUTNAME]} {
  BinVelocities $INPUTNAME.restart.vel
  BinCoordinates $INPUTNAME.restart.coor
} else {
  temperature $ITEMP
}

source mdff_template.namd
```

3. Executing MDFF simulation

We executed the following commands sequentially. Parameters in the NAMD configuration file of minimization (target_Xmini.namd) was same with target_Nmini.namd.

```
#Refinement with map potential of C-alpha atom positions
./charmrun ++local +p24 namd2 target_Xca.namd
#Refinement with map potential of backbone atom positions
./charmrun ++local +p24 namd2 target_Xbb.namd
#Refinement with map potential of all atom positions
./charmrun ++local +p24 namd2 target_Xall.namd
#Minimization with map potential of all atom positions
./charmrun ++local +p24 namd2 target_Xmini.namd
```
